# Do Not Attempt Resuscitation (DNAR) Orders in an Older-Age Psychiatric Hospital

**DOI:** 10.1192/bjo.2024.639

**Published:** 2024-08-01

**Authors:** Sian Thompson, Michael Whybrow

**Affiliations:** 1 Northern Care Alliance, Manchester, United Kingdom; 2 Royal Bolton NHS Trust, Bolton, United Kingdom

## Abstract

**Aims:**

We aim to see whether DNAR discussions are being undertaken at an appropriate time for our patients, as well as seeing whether these are recorded formally and regularly reviewed, as per local protocol. We also aim to see whether the immediate medical/nursing teams are aware of the local guidelines, as well as which of their patients have a DNAR in situ, and how to find this out. As an old-aged psychiatric unit, this is very important.

**Methods:**

We used 2 methods of data collection. One was questionnaires that we gave out to medics, nurses, and HCAs on our wards. We collected quantitative data from them on whether they knew where DNAR forms were and which of their patients had DNAR forms. We then also collected quantitative data from our online notes, looking into which patients had DNARs, whether these were recorded online and in a physical copy, whether it was discussed on clerking, and whether it was regularly reviewed and documented in MDTs. We used data from 51 inpatients over 3 wards.

**Results:**

Over 30% of patients have a DNAR in situ across the 3 wards. The dementia-focussed wards have a higher number of DNARs in place. All patients with a DNAR had a purple form completed and kept on the ward. 75% of staff knew where these were. Only 20% of those with DNARs had these documented online as per local guidelines; only 45% of staff knew where to find this information online. Only 8% of patients had their DNAR status discussed on admission, and 10% in their first MDT. Only 60% staff knew which patients had a DNAR in situ.

**Conclusion:**

There is evidence that purple forms are completed appropriately and stored well. The main issue is the online record-keeping; staff either don't know how to or that they can document this online. This is reiterated as many did not know where the information was online. This demonstrates a lack of knowledge and education.

DNAR conversations are not occurring in the first place; the status is not being regularly reviewed, leading to issues where these conversations are rushed during acute events. It is important to think about these things earlier to ensure everyone, patient, family and staff, understands the process and rationale.

Lack of staff knowledge on which patients have DNARs in situ could be a great issue if an acute event were to occur, and compromises patient safety.

